# ASSOCIATIONS BETWEEN CARDIORESPIRATORY FITNESS, BRAIN AGE, AND NEURODEGENERATION AMONG OLDER ADULTS

**DOI:** 10.1093/geroni/igae098.2304

**Published:** 2024-12-31

**Authors:** Grace Derboghossian, Keenan Pituch, Mia Anthony, Dereck Salisbury, Feng Vankee Lin, Fang Yu

**Affiliations:** Arizona State University, Phoenix, Arizona, United States; Arizona State University, Phoenix, Arizona, United States; University of Rochester, Rochester, New York, United States; University of Minnesota, University of Minnesota, Minnesota, United States; Stanford University School of Medicine, Stanford, California, United States; Arizona State University, Phoenix, Arizona, United States

## Abstract

There is growing evidence that CRF mitigates the likelihood of dementia caused by Alzheimer’s disease (AD) and may underlie the cognitive benefits observed from aerobic exercise. Previous evidence further demonstrates neurodegeneration is the biological substrate for cognition deterioration and brain age may protect the brain from the deleterious effects of neurodegeneration. However, little is known about the relationships between CRF, brain age, and neurodegeneration in older adults with amnestic mild cognitive impairment (aMCI). The purpose of this cross-sectional study was to examine associations between CRF, brain age, and neurodegeneration among individuals with aMCI, using baseline data from the Aerobic exercise and Cognitive Training Trial, which examines the cognitive effects and underlying mechanisms of a 6-month ACT in older adults with aMCI. CRF was measured with VO2peak from a symptom-limited peak cycle-ergometer test. Brain age, hippocampal volume, and AD-signature cortical thickness (SCT) were obtained from structural magnetic resonance imaging. Multiple linear regressions were conducted in R (version 4.3.2). The sample (N=134) averaged 73.63 ± 5.81 years of age, 16.98 ± 2.9 years of education, 27.47 ± 5.17 in BMI, and 23.5 ± 2.18 in Montreal Cognitive Assessment scores with 51.5% male and 92.5% White. The mean brain age was 72.37±7.76 years with 2.92± 0.31mm ADSCT and 3164 ±455.51mm3 hippocampal volume. CRF was not associated with brain age and neurodegeneration. A negative association was found between brain age with hippocampal volume (β=-11.37, SE=5.11, p=0.02) and ADSCT (β=-0.01, SE=0.003, p=0.004). To conclude, future studies need to explore other brain indicators related to CRF.

